# Reviewed article: Santos BRA, Oliveira GA, Silva GD, Santos JBR, Morgado M, Junior AAG, Acurcio FA, Alvares-Teodoro J. Evaluation of the performance of biological drugs in the treatment of ankylosing spondylitis: cohort study, Minas Gerais, 2018-2023. Epidemiol Serv Saude. 2025:34;e20240116.

**DOI:** 10.1590/S2237-96222025v34e20240116.b

**Published:** 2025-09-08

**Authors:** Christine Faustino

**Affiliations:** 1 UEMS, Medicine

**Table:** 

Rating	Excellent	Good	Average	Below average	Poor
Interest		✓			
Quality			✓		
Originality			✓		
Overall			✓		

**Table:** 

Questionnaire	Yes	No	Not applicable
Does the manuscript contain new and significant information to justify publication?	✓		
Does the Abstract (Summary) clearly and accurately describe the content of the article?		✓	
Is the problem significant and concisely stated?	✓		
Are the methods described comprehensively?	✓		
Are the interpretations and conclusions justified by the results?		✓	
Is adequate reference made to other work in the field?		✓	

## Manuscript Structure

Length of article is: Adequate

Number of tables is: Adequate

Number of figures is: Adequate

Please state any conflict(s) of interest that you have in relation to the review of this paper (state “none” if this is not applicable): None

## Comments

O tema é relevante, porém o texto emprega linguagem pouco clara, feito a partir de construções longas e sem observar as instruções aos autores. A seguir, são apontados os itens a serem aprimorados.

Itens críticos

1. O texto apresenta similaridade maior que 47%. Existem trechos com alta similaridade provenientes da referência “repositorio.ufmg.br” e neste caso, o texto deverá ser reescrito. Certifique-se que paráfrases e citações estejam de acordo com as normas da revista.

2. Título: pode ser reduzido. Revise o título do trabalho para informar o tema principal, delineamento, local e ano(s) da pesquisa; deve seguir as normas da revista (sem siglas, nome do estado por extenso etc.) e do guia de redação desse delineamento.

3. Sugere-se rever o título do eixo Y do gráfico, pois não se tratar de sobrevivência acumulativa, mas de continuação do tratamento, cautela na interpretação no valor de HR, pois de acordo com o gráfico o risco se altera após 30 meses e a inclusão abaixo do gráfico sobre tamanhos de amostra em cada um dos grupos.

4. O resumo precisa ser reescrito. A seção método não cita a fonte dos dados. Prepare versão da conclusão do seu resumo que responda com clareza ao objetivo. Essa conclusão deve se deter aos resultados que constam no resumo.

Itens importantes

1. Sugere-se que apenas o desfecho de descontinuação seja abordado no artigo, pois a discussão dos resultados relacionados a esse desfecho e persistência tornam a leitura confusa.

2. É preciso corrigir erros de concordância verbal, como por exemplo “e investigar a descontinuação e persistência associada”. Rever o emprego de letras maiúsculas em nomes que não são próprios. Verifique o uso de maiúsculas de acordo com as regras da língua portuguesa e remova de casos não pertinentes.

3. Verifique o número de casas decimais e aplique arredondamento pertinente: 1 casa decimal para percentual, 2 para medida de associação e 3 para p-valor.” “Empregue ‘<0,001’ quando for menor que 0,001, que deve substituir ocorrências ‘0,000’. Verifique a apresentação de dados externos na introdução e discussão, os quais não devem conter casas decimais, a menos que essa precisão seja realmente necessária.

4. Substitua construções como ‘Outros autores’, ‘Outros estudos’ e ‘A literatura aponta’ por dados objetivos do estudo citado, com a referência citada próxima a essa afirmação.

5. É necessário justificar o motivo pelo qual apenas os dados dos pacientes de Minas Gerais foram analisados.

6. Evite o uso de ‘respectivo’ ou ‘respectivamente’. Reformule a redação para trazer os dados próximos da sua correspondência, sem risco de confusão na leitura.

7. O coeficiente de Gini não costuma ser empregado para a avaliação da desigualdade de renda de um indivíduo, mas de municípios, regiões ou países. Isso precisa ser esclarecido na redação do método. Solicita-se que seja mencionada a fonte da qual o dado para os municípios foi coletado.

8. Na linha 17, página 4, é necessário substituir o ponto por vírgula.

9. Nas linhas 7 e 8 da página 6 afirma-se que o “Os resultados deste estudo demonstram que, na população brasileira, a doença tem acometimento em ambos os sexos”. Essa afirmação não pode ser realizada, pois foram analisados os dados apenas dos pacientes de MG.

10. Adequar as colunas de número absoluto e proporção de acordo com as normas da revista (trata-se de apenas uma coluna ao invés de duas).

11. A porcentagem de informações indisponíveis deve ser relatada na seção “Resultados”. Orienta-se que novos dados não sejam apresentados na discussão (página 6, linhas 16 e 17).

12. É necessário que as limitações do estudo sejam relatadas.

13. Incluir referências ao final ao final de algumas frases, como por exemplo nas linhas 50 a 59 da página 7 e 25 a 36 da página 8, pois existem artigos e outros materiais sobre o tema.

14. Os títulos das tabelas devem ser genéricos. Sugere-se consulta a artigos publicados recentemente na revista para verificação de exemplos de títulos.

15. Títulos de figura não devem incluir o tipo de gráfico. 

